# A Novel Immune-Related Seventeen-Gene Signature for Predicting Early Stage Lung Squamous Cell Carcinoma Prognosis

**DOI:** 10.3389/fimmu.2021.665407

**Published:** 2021-06-11

**Authors:** Tao Fan, Zhiliang Lu, Yu Liu, Liyu Wang, He Tian, Yujia Zheng, Bo Zheng, Liyan Xue, Fengwei Tan, Qi Xue, Shugeng Gao, Chunxiang Li, Jie He

**Affiliations:** ^1^ Department of Oncology, Renmin Hospital of Wuhan University, Wuhan, China; ^2^ Department of Thoracic Surgery, National Cancer Center/National Clinical Research Center for Cancer/Cancer Hospital, Chinese Academy of Medical Sciences and Peking Union Medical College, Beijing, China; ^3^ Department of Pathology, National Cancer Center/National Clinical Research Center for Cancer/Cancer Hospital, Chinese Academy of Medical Sciences and Peking Union Medical College, Beijing, China

**Keywords:** early stage lung squamous cell carcinoma, immune signature, gene mutation, immune cell infiltration, prognostic model

## Abstract

With the increasingly early stage lung squamous cell carcinoma (LUSC) being discovered, there is an urgent need for a comprehensive analysis of the prognostic characteristics of early stage LUSC. Here, we developed an immune-related gene signature for outcome prediction of early stage LUSC based on three independent cohorts. Differentially expressed genes (DEGs) were identified using CIBERSORT and ESTMATE algorithm. Then, a 17-immune-related gene (RPRM, APOH, SSX1, MSGN1, HPR, ISM2, FGA, LBP, HAS1, CSF2, RETN, CCL2, CCL21, MMP19, PTGIS, F13A1, C1QTNF1) signature was identified using univariate Cox regression, LASSO regression and stepwise multivariable Cox analysis based on the verified DEGs from 401 cases in The Cancer Genome Atlas (TCGA) database. Subsequently, a cohort of GSE74777 containing 107 cases downloaded from Gene Expression Omnibus (GEO) database and an independent data set consisting of 36 frozen tissues collected from National Cancer Center were used to validate the predictive value of the signature. Seventeen immune-related genes were identified from TCGA cohort, which were further used to establish a classification system to construct cases into high- and low-risk groups in terms of overall survival. This classifier was still an independent prognostic factor in multivariate analysis. In addition, another two independent cohorts and different clinical subgroups validated the significant predictive value of the signature. Further mechanism research found early stage LUSC patients with high risk had special immune cell infiltration characteristics and gene mutation profiles. In conclusion, we characterized the tumor microenvironment and established a highly predictive model for evaluating the prognosis of early stage LUSC, which may provide a lead for effective immunotherapeutic options tailored for each subtype.

## Introduction

With an estimated 228,820 new cases and 135,720 death cases in 2020, lung cancer is the most common cancer and a leading cause of cancer-related deaths worldwide (1). Accounting for approximately 30% of lung cancer, lung squamous cell carcinoma (LUSC) is one of most common lung cancer types (2). Commonly located in the central lung, LUSC frequently invades large blood vessels and proximal bronchus (3). Although the treatment of cancer has made great progress, including the improvement of surgical instruments and surgical methods and the discovery of new treatment methods and drugs such as chemotherapy, immunotherapy, and biological therapy, the average 5-year survival rate of lung cancer patients in most countries is only 10–20% (4, 5). The high mortality rate of lung cancer is attributed to the fact that patients are diagnosed at an advanced stage, and the lack of a good individualized treatment plan makes the treatment of lung cancer less than ideal. In recent years, with the improvement of people’s health awareness and the popularization of low-dose computed tomography (CT) for lung cancer screening, increasingly early stage lung cancers have been discovered. There is an urgent need to find more effective solutions for individualized treatment of early stage lung cancer.

In the past few years, scholars have conducted extensive research on the complex interaction between solid tumors and host immunity, but they were still poorly understood. It has been proved that the tumor-infiltrating immune cells were closely related to the prognosis of patients (6–13). For example, stimulation of tumor-infiltrating CD8 T cells enabled sustained antitumor responses (14). The combination of signal transducer and activator of transcription 3 (STAT3) inhibition and whole-brain radiation therapy (WHRT) could induce dendritic cell and T cell interactions to enhance the therapeutic effect against glimas (15). The infiltration of immune cells and abnormal expression of immune genes plays an irreplaceable role in the occurrence and progression of lung cancer. Th9 and Th17 lymphocytes promoted lung cancer cell migration and metastatic spreading by inducing tumor cell epithelial–mesenchymal transition (EMT) (16). Tumor-infiltrating B cells could efficiently present antigen to tumor-infiltrating CD4 T cells and alter the CD4 cells’ phenotype using an antigen-presentation assay, which further served as a potential therapeutic target in lung cancer immunotherapy (17). Innate and adaptive immune response participated in all aspects of antitumor and immune escape in the tumor microenvironment. The emergence of immune checkpoint inhibitors provides unprecedented opportunities for tumor immunotherapy. Recent breakthrough in immunotherapy was immune checkpoint programmed death 1 (PD-1)/programmed death ligand 1 (PD-L1) blockage in lung cancer, which has achieved great clinical successes (18). As the underlying mechanism of intrinsic and acquired resistance to lung cancer chemotherapy or PD-1/PD-L1 blockade therapy was still unclear; only about 20% of patients benefitted from treatment with immune checkpoint inhibitors (19, 20). Although some scholars have used immune-related genes to predict the prognosis of lung cancer before, they did not further use related genes to construct models and explore the mechanism.

Gene mutation is one of the most important internal factors for tumorigenesis. Jeong and his colleagues verified that the radio resistance of patients with LUSC was closely related to KEAP1/NRF2 mutations (21). The homozygous inactivation of LKB1 showed a strong cooperation with KRAS mutation in non-small cell lung cancer (NSCLC) (22). However, there are few studies on the relationship between immune-related genes and mutation-related gene expression in LUSC.

At present, the detection rate of early stage LUSC is soaring, and immunity plays an important role in tumorigenesis and progression. We need to study the characteristics of tumor microenvironment more deeply and find reliable tumor prognosis models. It is urgent to find new methods that could be used to prevent and treat tumor occurrence and progression based on related genes or models. In this study, we established an immune gene-related and individualized tumor prognosis model based on the whole genome sequencing data of 401 patients with early stage LUSC from the TCGA database. Furtherly, this predictive model was validated by a set of GEO data and an independent cohort of 36 frozen tissue samples. We also further explored the possible mechanism of the model for predicting the prognosis of early stage LUSC by analyzing the correlation between the immune-related risk genes and TME tumor-infiltrating immune cells or LUSC related mutation genes. Our research will help clarify the correlation and underlying mechanisms between immune-related genes and the prognosis of early stage LUSC and provide a basis for optimizing the immunotherapy of LUSC.

## Materials and Methods

### Study Design

**Figure 1:**
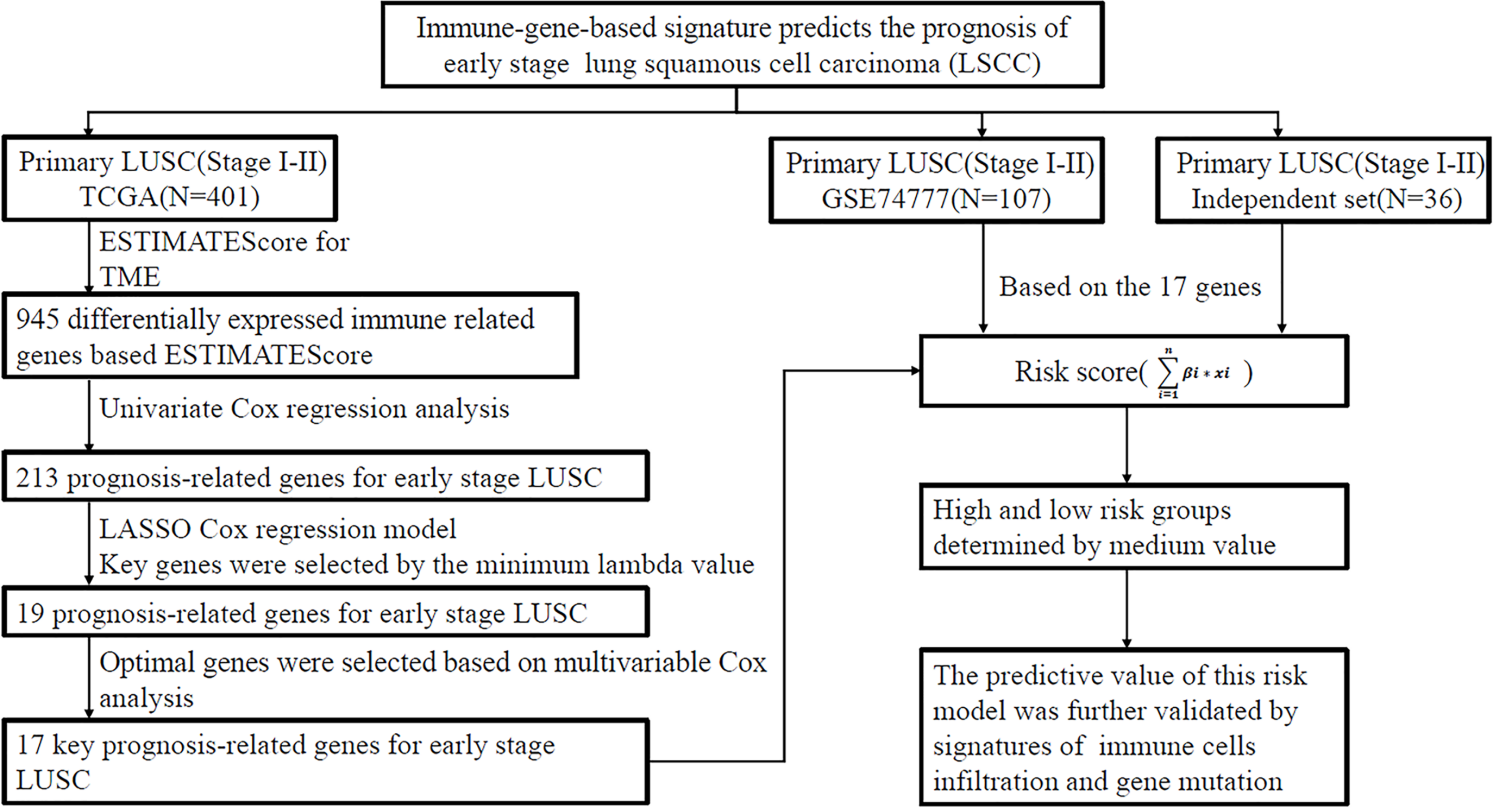
Analysis flow chart showing the screening process. The three cohorts including TCGA data set, GSE74777 data set (GEO), and independent cohort (National Cancer Center). LUSC, lung squamous cell carcinoma; TCGA, The Cancer Genome Atlas Program; GEO, gene expression omnibus database; LASSO least absolute shrinkage and selection operator; ESTIMATE, Estimation of STromal and Immune cells in MAlignant Tumor tissues using Expression data.

We aimed to develop an immune-related gene signature for outcome prediction of early LUSC based on three independent cohorts. Clinical data of 401 patients with LUSC were obtained from the TCGA database, which was acted as a training set and used to establish a risk model. A cohort of 107 cases (GSE74777) downloaded from GEO database and an independent set consisting of 36 frozen tissues collected from the National Cancer Center were used to validate the predictive value of this signature. The study pipeline was shown in **Figure 1**. This was a retrospective study and informed consent was waived by the Institutional Review Board in our hospital.

### Public Datasets

These results were generated by two publicly available datasets. RNA-seq data samples from 401 cases of early stage LUSC were downloaded from TCGA (https://portal.gdc.cancer.gov/), which included the corresponding clinical information of the patient. An independent validation sequencing data of 107 cases of early stage LUSC obtained from GSE74777 was downloaded from Gene Expression Omnibus (GEO) (https://www.ncbi.nlm.nih.gov/gds) database containing complete clinical information.

### Signature Generation and Predictive Model Construction

Tumor microenvironment score was calculated by ESTIMATE algorithm using “estimate” package in R software (version 3.6.1). The early stage LUSCs were classified into high-risk group and low-risk group by the median value of the ranked immune/stromal score. The DEGs were identified based on high or low score with the threshold of p value <0.05 and |log2 (fold change)| >2. Univariate Cox regression analysis was conducted on DEGs to identify a set of candidate prognostic genes. LASSO (least absolute shrinkage and selection operator) regression analysis with standard of one standard error (SE) and 100-fold cross-validation was performed to select the most important immune-related genes. Finally, the risk model was calculated based on the expression data of optimized genes and multivariate coefficients. The formula was as follows:

$$\text{risk score}=\text{coef}(1)*\text{gene}{\left(1\right)}_{\text{expr}}+\text{coef}(2)*\text{gene}{\left(2\right)}_{\text{expr}}+\ldots +\text{coef}(\text{n})*\text{gene}{\left(\text{n}\right)}_{\text{expr}}$$

According to the median of risk score, patients with early stage LUSC were divided into high-risk group and low-risk group. Kaplan–Meier and log-rank test were used to evaluate the over survival (OS) difference between high- and low-risk groups. *P*< 0.05 was considered statistically different.

### Immune Cell Infiltration Analysis

Characterized by 547 genes, the immune cell infiltration ratio was calculated by a deconvolution algorithm, which was widely reported as CIBERSORT (23). In our study, this algorithm was used to assess the relative proportion of 22 infiltrating immune cell types based on gene expression matrix. A 100× permutation count and *P <*0.05 were considered the threshold.

### Functional Enrichment of Identified DEGs

In order to study the potential mechanism of this model for predicting the prognosis of early stage LUSC, functional annotations of the immune-related genes were analyzed using R package “clusterProfiler”. Gene Ontology (GO) and Kyoto Encyclopedia of Genes and Genomes (KEGG) enrichment analysis including biological processes (BP), molecular functions (MF), and cellular components (CC) showed the molecular biological characteristics of patients with early stage LUSC.

### Patients and Tissue Samples

We obtained frozen tumor tissue from 36 patients with early stage LUSC who underwent radical surgery for lung cancer in the Chinese Academy of Medical Sciences/Cancer Hospital from January 2012 to December 2012. The total RNA was extracted from indicated tissues using trizol reagent according to the manufacturer’s protocol. A prime Script RT reagent kit (China, AQ601-01) was used to reverse transcribe total RNA samples to single-stranded cDNA, which was further prepared for q-PCR. **Supplementary Table 1** showed the primer sequences of the 17 genes.

### Statistical Analysis

Mann–Whitney *U* tests or Wilcoxon signed-rank was used to compare gene expression profiles. The Cox, tumor microenvironment, gene difference, and clinical characteristics analysis were conducted using packages implemented in R (v. 3.6.1). Survival analyses were performed by Graphpad Prism 8. The correlations between the risk score and immune genes were validated using “ggpubr” and “limma” R packages. *P <*0.05 was considered to be significant statistically The correlation of two variables was evaluated by Pearson’s correlation test. Mann–Whitney *U* test was used to compare the proportion of immune cell infiltration between high-risk group and low-risk group. The comparison of gene mutation rate between high-risk group and low-risk group was performed using Pearson chi-square test (T ≥5), or Yates’ continuity corrected chi-square test (1 ≤T <5), or Fisher’s exact test (T <1). R value and *p <*0.05 were considered to be the criteria for judging the existence of correlation.

## Results

### Establishment of Prognostic Risk Model for Patients With Early Stage LUSC in TCGA Cohort

**Figure 2:**
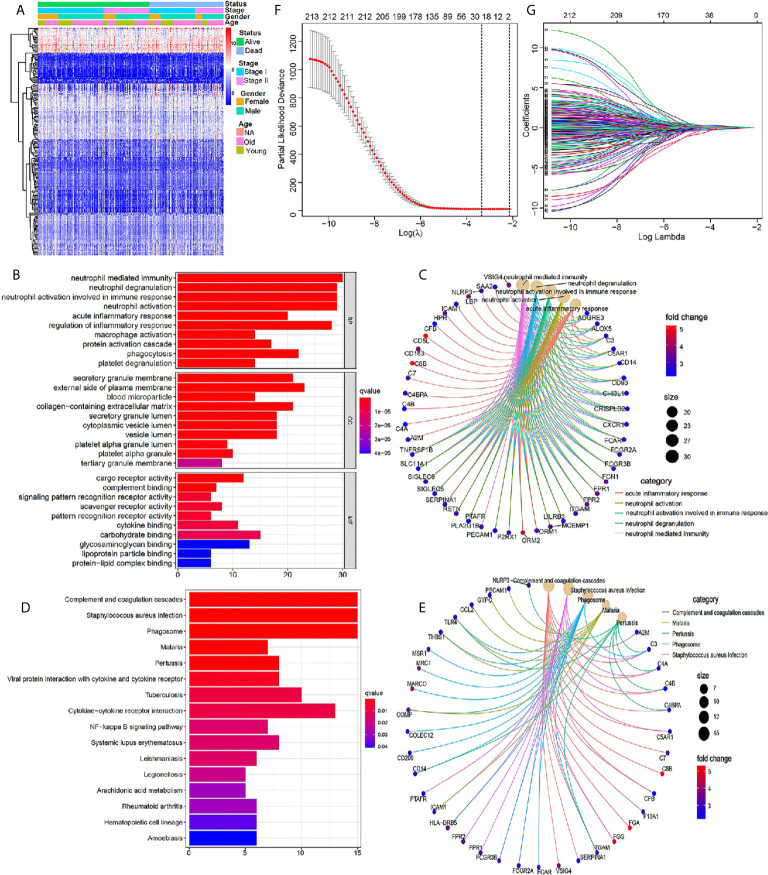
Identification of core prognostic differentially expressed immune-related genes. **(A)** Heatmap showing gene expression value across each sample, and the distribution of status by patient gender, TNM stage and age. **(B, C)** GO analysis of the core prognostic differentially expressed immune-related genes. **(D, E)** KEGG analysis of the identified prognostic genes. **(F)** LASSO model selecting most useful parameter. **(G)** LASSO coefficient graph of the key prognostic immune-related genes.

We scored the tumor immune microenvironment of 401 cases of early stage LUSC from the TCGA database, and the results showed that there were 945 DEGs between high-immune (stromal) score group and low- (stromal) score group with the threshold of p value <0.05 and |log2 (fold change)| >1 (**Supplementary Figure 1**). Further GO and KEGG enrichment analyses demonstrated that these DEGs were closely related to immune signaling pathway (**Supplementary Figure 2**). Further univariate Cox regression analysis was used to identify 213 immune-related genes, which were closely related to the patient’s prognosis (*P <*0.05) (**Supplementary Table 2**). A heatmap indicated the detailed expression signature of the 213 immune-related genes (**Figure 2A**). GO and KEGG enrichment analyses for these 213 genes were conducted to confirm functions of these key genes in tumorigenesis and progression. The biological processes revealed that these candidate immune-related genes were particularly involved in “neutrophil mediated immunity”, “neutrophil degranulation”, “neutrophil activation involved in immune response”, “neutrophil activation”, “acute inflammatory response”, and “regulation of inflammatory response” (**Figure 2B**). **Figure 2C** showed the main genes regulating these top five biological processes. KEGG enrichment analysis indicated that these immune-related genes were involved in “complement and coagulation cascades” and “*Staphylococcus aureus* infection” (**Figure 2D**). **Figure 2E** showed the main genes regulating these top five pathways enriched by KEGG. All the above results revealed that these prognostic genes were closely related to immune response and inflammation signaling pathway. Next, 19 most important prognostic immune-related genes (RPRM, APOH, HPR, SSX1, MSGN1, CCDC177, ISM2, FGA, LBP, HAS1, CSF2, RETN, CCL2, CCL21, MMP19, PTGIS, F13A1, C1QTNF1, ODAPH) were screened out by a LASSO COX regression analysis with standard of one standard error (SE) and 100-fold cross-validation (**Figures 2F, G** **)**.

**Figure 3:**
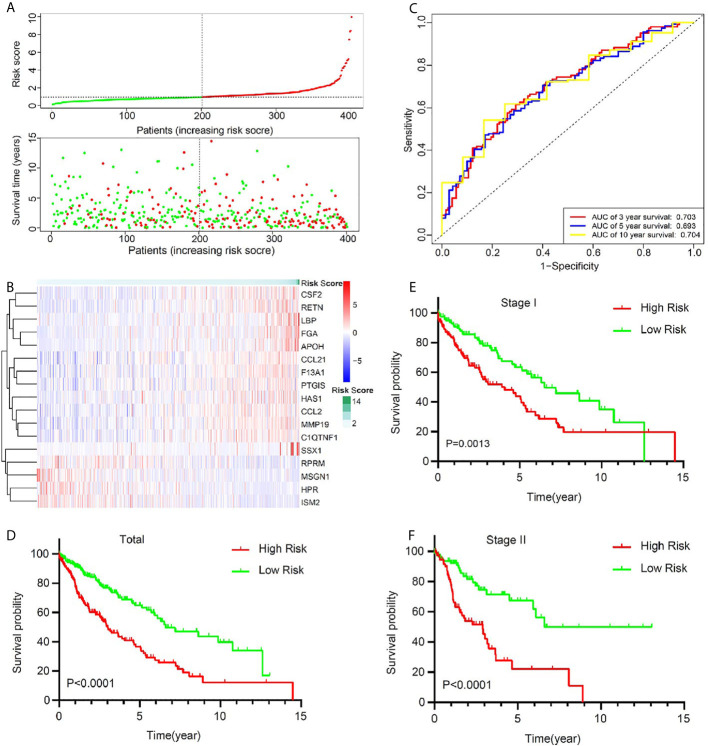
The signature of key prognostic immune-related genes and their potential predictive value in early stage LUSC based on a TCGA training set. **(A)** The distribution of risk score and survival status. **(B)** Distribution of specific gene expression based on risk score. **(C)** ROC curves of these key genes to predict death risk at 3, 5, and 10 years in TCGA set. **(D)** Comparison of overall survival of all patients with early stage LUSC between high risk and low risk. **(E, F)** Kaplan–Meier curves of patients with stage I and stage II LUSC based on risk score.

To optimize this model, we finally identified 17 genes (RPRM, APOH, SSX1, ISM2, HPR, MSGN1, FGA, LBP, HAS1, CSF2, RETN, CCL2, CCL21, MMP19, PTGIS, F13A1, C1QTNF1) to build the risk score by a stepwise Cox proportional hazards regression analysis (**Supplementary Table 3**). In TCGA cohort, all the 401 patients were divided into high-risk group and low-risk group according to the median risk value (**Figure 3A**). A heatmap showed and compared the 17 genes’ expression signature between the high-risk group and the low-risk group (**Figure 3B**). The ROC curves were used to evaluate predictive value of this model (**Figure 3C**), which showed a good predicted accuracy with the 3-year (AUC = 0.703), 5-year (AUC = 0.693), and 10-year (AUC = 0.704) overall survival (OS). Patients with high risk presented a low survival possibility (**Figure 3D**) (*P* < 0.05). To further confirm predictive value of this model, we compared the survival curves of patients with high- and low-risk scores in each subgroup. As was shown in **Figures 3E, F**, high-risk scores among LUSC patients with stage I or stage II were presented with a poor OS (*P* < 0.05). Similarly, the mortality rate in high-risk group was significantly high than that in low-risk group among female or male patents (**Supplementary Figures 3A, B** **)**, and a same trend appeared in patients with age ≤65 or age >65 (**Supplementary Figures 3C, D** **)**. Current smoker or current reformed smoker with high risk presented a poor OS except for lifelong non-smoker (**Supplementary Figures 3E–H**).

### Validation of the Risk Model in GEO Dataset

**Figure 4:**
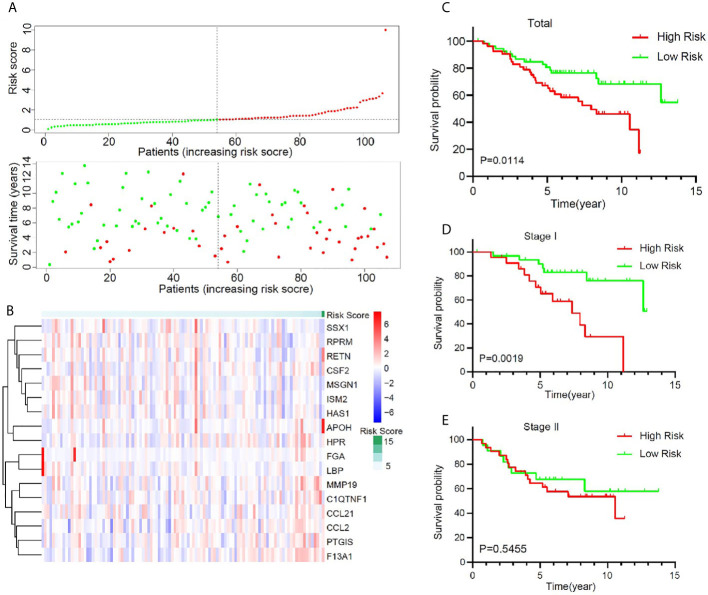
Validation of the prognostic value of the key immune-related gene signature in GSE74777. **(A)** The distribution of risk score and survival status. **(B)** Distribution of specific gene expression based on risk score. **(C)** Comparison of overall survival of all patients with early stage LUSC between high risk and low risk. **(D, E)** Kaplan–Meier curves of patients with stage I and stage II LUSC based on risk score.

To validate the predictive value of this model, an independent cohort GSE74777 was downloaded from GEO. A total of 107 early stage LUSC patients were divided into a high-risk group (n = 53) and a low-risk group (n = 54) using the same method (**Figure 4A**). A heatmap showed and compared the 17 genes’ expression signature between the high-risk group and the low-risk group (**Figure 4B**). Patients with high risk presented a low survival possibility (**Figure 4C**) (*P* < 0.05). To further confirm the predictive value of this model, we compared the survival curves of patients with high- and low-risk scores in each subgroup. As was shown in **Figure 4D**, high-risk score among patients with stage I was presented with a poor OS (*P* < 0.05). However, OS showed no significant difference in stage II patients between high- and low-risk score, which may be attributed to the limited sample size (**Figure 4E**, *P* > 0.05). In subgroups, including male, age >65, and non-smoker, a similar result was confirmed (**Supplementary Figure 4**, *P* < 0.05).

### Tumor-Infiltrating Immune Cells Landscape of Early Stage LUSC With Different Risk Scores

**Figure 5:**
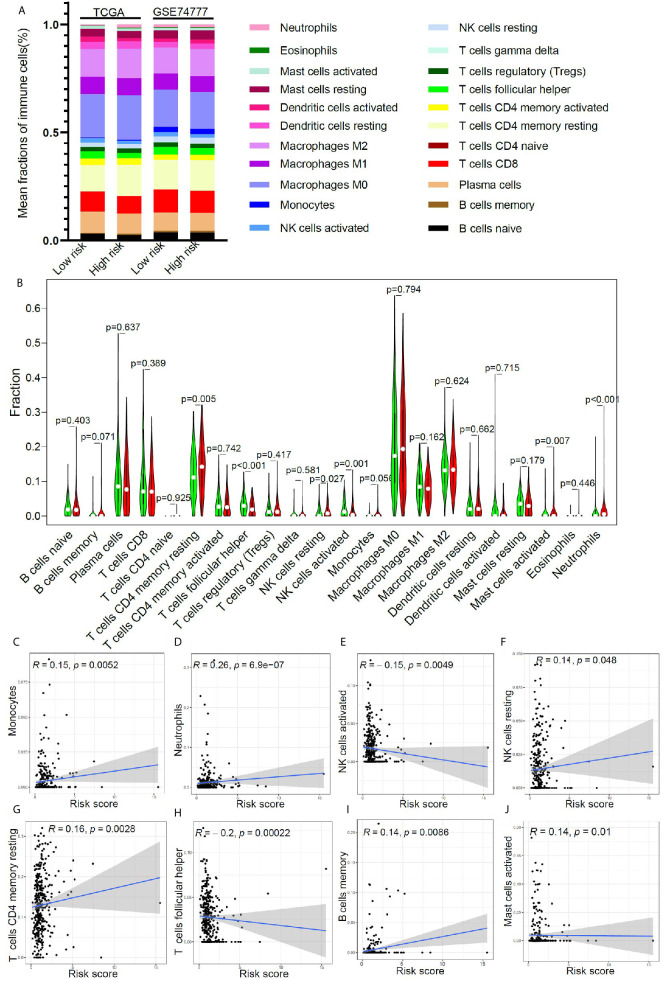
The relationship between immune cell infiltration and risk score in early stage LUSC. **(A)** The distribution of tumor immune cell infiltration in high- and low- risk groups in TCGA cohort and GSE74777 set. **(B)** Comparison of tumor immune cell infiltration levels between high- and low-risk groups. **(C–J)** Correlation of risk score with immune cell infiltration levels in patients with early stage LUSC.

In order to further clarify the potential mechanism of this model, based on 17 immune-related genes, that could be used to predict the prognosis of early stage LUSC, we further studied the relationship between the risk score and immune cell infiltration in the tumor microenvironment. Using CIBERSORT algorithm, we got the relative proportion of immune cell infiltration. The estimated distribution of 22 immune cell types in high-risk group and low-risk group was presented in **Figure 5A**. We further compared the differences in the distribution of immune cells in the high- and low-risk groups. As shown in **Figure 5B**, the infiltration proportion of T cells CD4 memory resting, NK cells resting, mast cells activated, and neutrophils in high-risk group was significantly higher than that in low-risk group, but the infiltration proportion of T cells’ follicular helper and NK cells activated in high-risk group was significantly lower than that in low-risk group. We further studied the correlation between the risk score and the proportion of immune cell infiltration. The result revealed that monocytes (**Figure 5C**), neutrophils (**Figure 5D**), NK cells resting (**Figure 5F**), T cells CD4 memory resting (**Figure 5G**), B cells memory (**Figure 5I**), and mast cells activated (**Figure 5J**) were positively correlated with the risk score, and NK cells activated (**Figure 5E**) and T cells’ follicular helper (**Figure 5H**) were negatively correlated with the risk score.

### Validation of the Risk Model Among Patients With Gene Mutation Status

**Figure 6:**
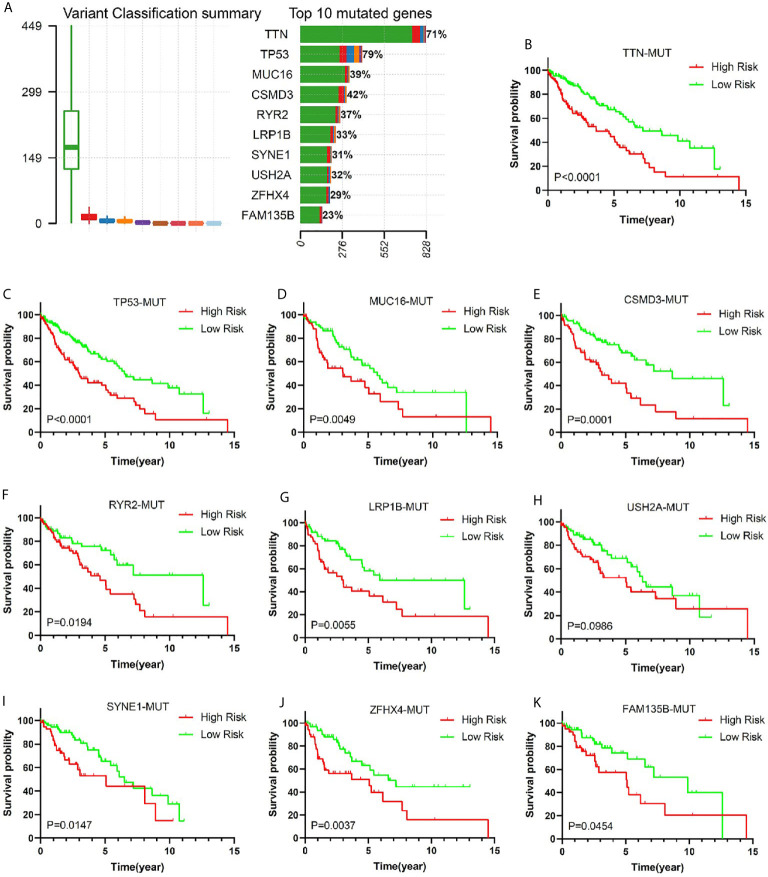
The relationship between risk score and common mutant genes in early stage LUSC patients. **(A)** The variant classification summary and top 10 mutated genes in LUSC. Kaplan–Meier curves of early stage LUSC patients with TTM mutation **(B)**, TP53 mutation **(C)**, MUC16 mutation **(D)**, CSMD3 mutation **(E)**, RYR2 mutation **(F)**, LRP1B mutation **(G)**, USH2A mutation **(H)**, SYNE1 mutation **(I)**, ZFHX4 mutation **(J)**, or FAM135B mutation **(K)** based on risk score.

Gene mutation is an important cause of LUSC. In order to verify the predictive value of this risk model in the population of patients with different genetic mutation types, we compared the OS of patients with low risk and high risk in different gene mutation status. Comprehensive analysis of nine gene mutation types (missense mutation, nonsense mutation, frame shift del mutation, splice site mutation, frame shift ins mutation, in frame del mutation, in frame ins mutation, translation start site mutation, and non-stop), found 10 genes (TTN, TP53, MUC16, CSMD3, RYR2, LRP1B, USH2A, SYNE1, ZFHX4, and FAM135B) with the highest mutation rate in LUSC patients (**Figure 6A**). It was very consistent that the early stage LUSC patients with one of the 10 genes’ mutation showed a worse prognosis in high-risk group compared with those in low-risk group (**Figures 6B–K**). Among wild-type status, overall survival was significantly longer in low-risk groups (**Supplementary Figure 5**).

To gain insight into the potential mechanism of predictive value of this model in early stage LUSC, we compared the top 10 genes’ mutation frequency of patients in different risk groups. The result showed that high-risk patients had a lower TTN, TP53, and CSMD3 mutation frequency (**Supplementary Figure 6**).

### Validation of the Risk Model in a Set of Independent Frozen Samples

**Figure 7:**
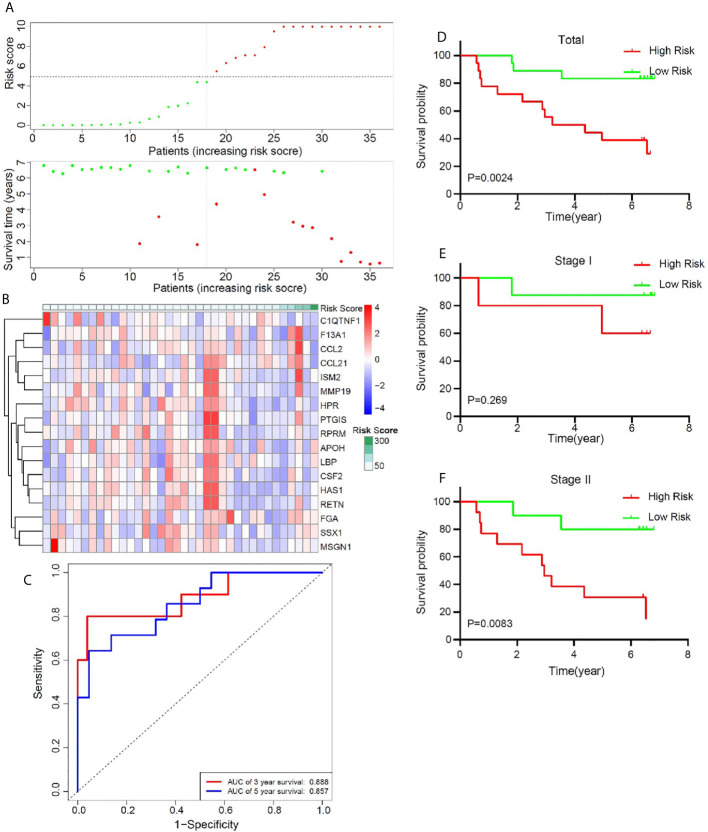
Validation of the prognostic value of the key immune-related gene signature based on a group of 36 frozen tissues from early stage LUSC patients. **(A)** The distribution of risk score and survival status. **(B)** Distribution of specific gene expression based on risk score. **(C)** ROC curves of these key genes to predict death risk at 3 and 5 years. **(D)** Comparison of overall survival of all patients with early stage LUSC between high risk and low risk. **(E, F)** Kaplan–Meier curves of patients with stage I and stage II LUSC based on risk score.

In order to better confirm the predictive value of this model in early stage LUSC, an independent cohort consisting of frozen tissue samples from 36 LUSC patients was used to validate this valuable signature. **Table 1** showed the detailed clinical characteristics of this group of patients. Based on a similar method, 36 early stage LUSC patients were divided into a high-risk group (n = 18) and the low-risk group (n = 18). The distribution of risk score, identified gene expression, and survival status were shown in **Figures 7A, B**. The ROC curves were used to evaluate predictive value of this model (**Figure 7C**), which showed a good predicted accuracy with the 3-year (AUC = 0.888) and 5-year (AUC = 0.857) overall survival. Patients with high risk presented a low survival possibility (**Figure 7D**) (*P* < 0.05). To further confirm predictive value of this model, we compared the survival curves of patients with high- and low-risk scores in each subgroup. As shown in **Figure 7F**, high-risk score among patients with stage II was presented with a poor survival rate (*P* < 0.05). In subgroups, including age ≤65 and smoking index ≤30, a similar result was confirmed (**Supplementary Figures 7A, C**, *P* < 0.05). However, overall survival rate showed no significant difference in patients with stage I, age >65, or smoking index >30 between high- and low-risk scores, which may be attributed to the limited sample size (**Figure 7E**, and **Supplementary Figures 7B, D** *P* > 0.05).

**Table 1:** Clinical characteristics of early stage lung squamous cell carcinoma.

Characteristics	TCGA cohort	GSE74777	Independent set
	N = 401	N = 107	N = 36
Age, year	67.58	64.54	60.53
mean			
Gender			
male	293	96	35
female	108	11	1
Current smoking			
Yes	110	54	32
No	280	53	4
NA	11	0	0
Stage			
I	242	54	13
II	159	53	23
Mutation status			
TTN MUT/WT	300/101	/	/
TP53 MUT/WT	303/98	/	/
MUC16 MUT/WT	172/229	/	/
CSMD3 MUT/WT	169/232	/	/
RYR2 MUT/WT	145/256	/	/
LRP1B MUT/WT	128/273	/	/
USH2A MUT/WT	133/268	/	/
SYNE1 MUT/WT	139/262	/	/
ZFHX4 MUT/WT	119/282	/	/
FAM1358 MUT/WT	101/300	/	/

### The Risk Score Based on These 17 Genes Was an Independent Risk Factor for Early Stage LUSC Patients

The comprehensive analysis and verification of these three independent cohorts showed that early stage LUSC patients with the high-risk score had a poor prognosis. In order to study whether the risk score was an independent prognostic factor for early stage LUSC, we performed univariate and multivariate Cox regression analyses. The results from TCGA indicated that the risk score based on these 17 genes could act as an independent predictor of OS (*P* < 0.001) after adjusting by T stage, N stage, TNM stage, age, sex, smoking history, TTN, TP53, MUC16, CSMD3, RYR2, LRP1B, USH2A, SYNE1, ZFHX4, and FAM135B mutation status (**Table 2**). Similar results were generated from GSE74777 (**Supplementary Table 4**, *P* = 0.01) and our independent cohort (**Supplementary Table 5**, *P* = 0.012), which all confirmed the predictive value of this risk model in patients with early LUSC.

**Table 2:** Univariable and multivariable Cox regression analyses of characteristics with overall survival in TCGA cohort.

Variable	Univariate cox			Multivariate cox		
	HR	CI95%	*P* Value	HR	CI95%	*P* Value
Age						
≤65 or >65	1.161	0.832–1.619	0.38	1.23	0.867–1.744	0.246
Gender						
Male or female	0.941	0.654–1.354	0.742	1.015	0.694–1.483	0.939
Current smoking						
Yes or no	1.44	1.03–2.013	0.033	1.573	1.099–2.251	0.013
T stage						
1, 2, or 3	1.25	0.941–1.66	0.124	1.293	0.905–1.849	0.159
N stage						
0, 1, or 2	0.905	0.629–1.301	0.589	0.906	0.491–1.672	0.752
TNM stage						
I or II	1.13	0.818–1.56	0.458	1.23	0.672–2.25	0.502
Mutation status						
TTN MUT or WT	0.522	0.371–0.734	<0.001	0.643	0.428–0.966	0.034
TP53 MUT or WT	0.84	0.578–1.221	0.36	1.039	0.698–1.546	0.851
MUC16 MUT or WT	1.061	0.774–1.455	0.711	1.645	1.147–2.36	0.007
CSMD3 MUT or WT	0.763	0.554–1.051	0.097	0.942	0.661–1.343	0.743
RYR2 MUT or WT	0.741	0.531–1.035	0.079	0.635	0.44–0.915	0.015
LRP1B MUT or WT	0.95	0.679–1.329	0.765	1.044	0.715–1.523	0.824
USH2A MUT or WT	0.774	0.553–1.084	0.136	0.813	0.567–1.166	0.26
SYNE1 MUT or WT	0.719	0.51–1.015	0.061	0.874	0.606–1.26	0.47
ZFHX4 MUT or WT	0.845	0.597–1.195	0.341	1.008	0.682–1.491	0.966
FAM1358 MUT or WT	0.681	0.464–1.001	0.051	0.815	0.542–1.226	0.326
Risk score						
Increasing	2.299	1.661–3.182	<0.001	2.396	1.679–3.419	<0.001

## Discussion

During the past ten years, many breakthroughs have been achieved in cancer research. The emergence of new therapeutic drugs and the combination of multiple disciplines have brought new hope for humans to overcome cancers. However, the OS of lung cancer has not improved significantly. With the popularization and application of low-dose CT, increasing patients are diagnosed with lung cancer at an early stage. In-depth understanding of early stage lung cancer and finding reliable prognostic-related genes are urgently needed, which can help us carry out necessary interventions for early stage lung cancer patients with potentially poor prognosis. Considering that the human immune system plays an important role in the occurrence and progression of LUSC (7, 24, 25), the establishment of a predictive model based on immune gene for early stage LUSC will be highly meaningful.

As we all know, with the development of various omics and the application of computer language in bioinformatics, molecular prediction models based on RNG-seq and chip data have been widely acquired and applied to predict patient prognosis. The proportion of immune cell infiltration calculated by computer simulation analysis was considered to be related to the prognosis of lung adenocarcinoma (LUAD) and lung squamous cell carcinoma (LUSC) (26). Fourteen immune-related genes have been identified to affect the prognosis of patients, which were further used to construct a signature to predict the prognosis of LUSC (27). In addition, Tang and his colleagues identified a set of mRNA expression prognostic signatures based on a large-scale meta-analysis (28). CX3CL1 was verified to have the potential function for predicting the prognosis of lung cancer, and this function may be attributed to its regulation of ‘positive regulation of cell adhesion’, ‘leukocyte cell–cell adhesion’, ‘leukocyte migration’ and ‘T cell activation’ (29). All the above studies have their own characteristics and new discoveries. Compared with these studies, our multi-cohort study was more comprehensive and more convincing and can better reflect the characteristics of the immune microenvironment. In our study, for the first time, we screen out candidate immune-related genes through the tumor microenvironment score and then use Cox regression analysis and LASSO regression analysis to select 17 target genes, which were furtherly used to conduct a risk model using the formula. Verified by three independent cohorts, it was confirmed that this risk model based on 17 immune-related genes was closely related to the OS of patients with early stage LUSC. More importantly, this model showed good predictive value in patients with early stage LUSC of different clinical subtypes and mutant subtypes. Furthermore, we further explored the potential mechanism of this model based on immune-related genes for predicting the prognosis of early stage LUSC, which laid a foundation for us to research the mechanism of immune cells, immune-related genes, and mutation-related genes in early stage LUSC from the level of transcriptomics in the future and provided new potential targets for immunotherapy of LUSC.

In this research, a 17 immune gene survival signature (including RPRM, APOH, SSX1, MSGN1, HPR, ISM2, FGA, LBP, HAS1, CSF2, RETN, CCL2, CCL21, MMP19, PTGIS, F13A1, C1QTNF1) was generated from 401 tumor samples of early stage LUSC. Most of these genes were involved in the proliferation, invasion, and differentiation of tumor cells (30–41). For example, wt-p53 downstream gene RPRM could be activated by HspB2 and further inhibited pancreatic cancer cell proliferation (42). It has been reported that the administration of FGA inhibited cell proliferation and migration and induced apoptosis in A549 cells (32). In the study of colorectal cancer (CRC) and hepatocellular carcinoma (HCC), it was found that the expression of APOH in tumor tissues was higher than that in adjacent tissues, and APOH showed a perfect function as a biomarker (43, 44). Synovial sarcoma X chromosome family member 1 (SSX1) promotes tumor cell metastasis and leads to tumor deterioration. The fusion of SS18 (SYT) and SSX1, SSX2, or SSX4 was reported to act as an oncogenic transcriptional regulator (45). SSX members were upregulated in various types of advanced cancers and in MHC class 1-deficient germline cells (46). Haptoglobin-related protein (HPR) interacted with lytic factor 2 (TLF2) and functioned as a ligand for a parasite receptor to play a role in antimicrobial immunity (47). Lipopolysaccharide binding protein (LBP) was a key serum molecule for TLR4 internalization, which could induce IFN-*β* expression and involved in tumor immunotherapy response (48, 49). Prostaglandin I2 synthase (PTGIS) was reported to promote the infiltration of tumor-associated macrophages (TAMs) and Tregs in TME, and high expression of PTGIS was associated with poor OS in multiple tumors (40). Chemokine CCL2 could induce the recruitment of M2-like tumor-associated macrophages and regulatory T cells, thereby coordinating the initiation of metastasis with immunosuppression and neovascularization (50). Chemokine CCL21 can induce the migration of antigen-presenting dendritic cells from the interstitium to the lymphatic system (51), and another study indicated that tenascin-C enhanced an immunosuppressive lymphoid stroma through CCL21/CCR7 signaling, leading to an increase in metastatic tumors (52). MSGN1, ISM2, HAS1, RETN, MMP19, C1QTNF1, and F13A1 were all involved in the regulation of tumors, but their involvement in the regulation of tumor immunity is not clear.

It was reported that the proportion of immune cell infiltration in tumor microenvironment was closely related to patient’s prognosis (53–57). For example, neutrophil infiltration in tumor environment was associated with a better prognosis (58). Triple-negative breast cancer patients with a high percentage of CD8+ cell infiltration had a higher long-term survival rate (59). To further clarify the mechanism of this model for predicting the prognosis of early LUSC, we evaluated the immune cell infiltration in the tumor microenvironment of patients with different risk score. In low-risk group, the infiltration proportion of T cells CD4 memory resting, NK cells resting, mast cells activated, and neutrophils was significantly reduced. In contrast, the infiltration proportion of T cells’ follicular helper and NK cells activated in low-risk group was significantly increased. Correlation analysis found that monocytes, neutrophils, NK cells resting, T cells CD4 memory resting, B cells memory, and mast cells activated were positively correlated with the risk score, and NK cells activated and T cells’ follicular helper were negatively correlated with the risk score. Based on the results of the difference analysis and correlation analysis, we found that patients in the low-risk group had better long-term survival rates, which may be due to the high proportion of NK cells activated and T cells follicular helper (60–62).

Most gene mutations were the initiating factor of tumorigenesis. More importantly, some gene mutations affected the prognosis and treatment effects of cancer patients. Recent studies have shown that gene mutations were closely related to the effect of tumor immunotherapy (63–66). In order to gain insight into the possible mechanism of this risk model for predicting the prognosis of patients with early stage LUSC, we studied the relationship between the risk score and the top ten genes with mutation frequency. The result demonstrated that patients’ mutation rate of TTN, TP53, and CSMD3 in high-risk group was significantly lower than that of patients in the low-risk group. As was reported, patients with TTN mutation showed longer OS or progression-free survival than those with wild-type status (67, 68). Recent study indicated that lung adenocarcinoma patient with TP53 missense mutant was linked to better clinical benefits taking antiPD-1/1L (69). In addition, La and his colleagues found CSMD3 mutation was associated with a better prognosis (70). Based on the above research reports and our study results, we speculated that the reason for the poor prognosis of patients in the high-risk group of this model may be related to TTN, TP53, and CSMD3 mutations.

In conclusion, we established a risk model based on 17 immune-related genes to predict the prognosis of patients with early stage LUSC, which was validated by three independent cohorts. By describing immune cell infiltration characteristics and gene mutation profiles, we clarified the potential mechanism of this model for predicting the prognosis of early LUSC, which would be useful to help clinicians implement individualized treatment for lung cancer patients, especially in tumor immunotherapy and individualized treatment of patients with special gene mutations.

## Data Availability Statement

The raw data supporting the conclusions of this article will be made available by the authors, without undue reservation.

## Ethics Statement

The studies involving human participants were reviewed and approved by the Ethics Committee of the Cancer Hospital of the Chinese Academy of Medical Sciences. No individual patient data was reported, and consent was not applicable for this study. Written informed consent for participation was not required for this study in accordance with the national legislation and the institutional requirements.

## Author Contributions

TF, YZ, LX, BZ, and CL designed the study, performed data collection, and conducted data analysis. TF, ZL, LW, YL, and HT drafted the manuscript. JH, CL, FT, QX, and SG revised and approved the paper. All authors contributed to the article and approved the submitted version.

## Funding

This work was supported by the National Key R&D Program of China (2018YFC1312100), the National Natural Science Foundation of China (81972196), The CAMS Innovation Fund for Medical Sciences (CIFMS) (2019-I2M-2-002).

## Conflict of Interest

The authors declare that the research was conducted in the absence of any commercial or financial relationships that could be construed as a potential conflict of interest.
