# Supplementary material for: A Novel Immune-Related Seventeen-Gene Signature for Predicting Early Stage Lung Squamous Cell Carcinoma Prognosis
Source: Front Immunol. 2021 Jun 11;12:665407. doi: 10.3389/fimmu.2021.665407 (PMC8226174; doi:10.3389/fimmu.2021.665407)
Supplement: Supplementary file 1 [file DataSheet_1.docx]

| **Supplementary Table 1. Primer sequences for qRT-PCR** | | |  |
| --- | --- | --- | --- |
| **Gene Name** | **Forward Primer** | **Reverse Primer** | |
| C1QTNF1 | AGGTGGTGATCTTGTTCGC | GTTCTCACGTTCGCCCTT | |
| RETN | GGAGTGCCAGAGCGTCA | ACATCCCACGAGCCACA | |
| RPRM | CTACTGACCCGCCCTCTG | TGACTCCGACAGGTTTGCT | |
| MMP19 | CGCTGGAGAAAGAAGCAC | GAAGGTCAAGGGAGCCA | |
| ISM2 | CATCAAGGTGGTGGAGGAC | CCCAGAGGAAGGACCAGA | |
| MSGN1 | TAGAGGTGGACTACAATATGTTAGC | TGAGTGTCTGGATCTTGGTGA | |
| HAS1 | TGCTGTGGGTGCTGCTG | TGTAGAGGGGCGCGTAGAG | |
| CCL2 | TGAAGCTCGCACTCTCG | GTGACTGGGGCATTGATT | |
| F13A1 | GCTGTGCTGGGAAAAGAC | AGAAGGTGATGTTGGCTGA | |
| PTGIS | CAGGGACAGAAGCAGGAG | CAGGCAGGTAGGGCATT | |
| CCL21 | CAGGCCAGGTCCAGAGAG | CAAGGAAGAGGTGGGGTGT | |
| FGA | GGGGCTCTGTTCTTAGGG | GGAGACTTGGAGGGCATAG | |
| APOH | ACGACACATGGAAATTGGA | AAGTGTTGGTTTTGCAGGA | |
| CSF2 | AACTTCCTGTGCAACCCA | CTCATCTGGCCGGTCTC | |
| HPR | GTCATTTCCCTCCTGCTCT | GCGAAACAAGTGCTCCA | |
| LBP | GGCATCAGCATTTCGGTCAAC | AACAGCCACCCCAAGTCTCCC | |
| SSX1 | AGGGTTTCCTTATGTTGGC | GGTGTCGTCTCCGTTCA | |

| **Supplementary Table 2. Prognostic associated gene list in early-stage LUSC from TCGA.** | | | | | | | |
| --- | --- | --- | --- | --- | --- | --- | --- |
| No. | Gene | HR | p value | No. | Gene | HR | p value |
| 1 | APOH | 1.6357 | <0.0001 | 31 | CCL21 | 1.1632 | 0.0029 |
| 2 | FGA | 1.2755 | <0.0001 | 32 | TMEM236 | 3.5037 | 0.0030 |
| 3 | HPR | 2.2084 | 0.0001 | 33 | MEDAG | 1.2684 | 0.0031 |
| 4 | FGG | 1.1581 | 0.0001 | 34 | PDE1B | 2.1049 | 0.0031 |
| 5 | RETN | 1.3818 | 0.0001 | 35 | PLA2G1B | 1.2868 | 0.0031 |
| 6 | LBP | 1.3631 | 0.0002 | 36 | SERPINA1 | 1.1534 | 0.0031 |
| 7 | TGM2 | 1.2530 | 0.0002 | 37 | F13A1 | 1.1928 | 0.0033 |
| 8 | C11orf96 | 1.2924 | 0.0003 | 38 | FAM107A | 1.2727 | 0.0035 |
| 9 | SLC22A3 | 1.2905 | 0.0005 | 39 | LYVE1 | 1.3542 | 0.0035 |
| 10 | FCN3 | 1.2364 | 0.0006 | 40 | VSTM1 | 2.3835 | 0.0038 |
| 11 | MCEMP1 | 1.2641 | 0.0009 | 41 | AKAP2 | 9.2607 | 0.0041 |
| 12 | NR4A3 | 1.3215 | 0.0010 | 42 | MMRN1 | 1.3692 | 0.0043 |
| 13 | AOC3 | 1.2610 | 0.0010 | 43 | CLIC5 | 1.3069 | 0.0044 |
| 14 | RRAD | 1.1942 | 0.0010 | 44 | CRISPLD2 | 1.2406 | 0.0047 |
| 15 | CCDC69 | 1.3363 | 0.0010 | 45 | CHIA | 1.6162 | 0.0048 |
| 16 | MMP19 | 1.2973 | 0.0011 | 46 | GKN2 | 1.3095 | 0.0050 |
| 17 | CSF2 | 1.4028 | 0.0011 | 47 | ASPA | 3.1299 | 0.0051 |
| 18 | DLC1 | 1.3697 | 0.0014 | 48 | SLC46A2 | 1.5261 | 0.0055 |
| 19 | FCN1 | 1.4417 | 0.0015 | 49 | ORM1 | 1.2076 | 0.0056 |
| 20 | SERTM2 | 1.8652 | 0.0016 | 50 | GFPT2 | 1.2550 | 0.0060 |
| 21 | SNAI1 | 1.3664 | 0.0017 | 51 | MYCT1 | 1.4376 | 0.0061 |
| 22 | ANKRD1 | 1.3006 | 0.0017 | 52 | ALOX5AP | 1.2103 | 0.0063 |
| 23 | FHL5 | 1.6066 | 0.0018 | 53 | CSF2RA | 1.3366 | 0.0065 |
| 24 | TCF21 | 1.4851 | 0.0019 | 54 | C7 | 1.1479 | 0.0066 |
| 25 | PTGIS | 1.2778 | 0.0024 | 55 | FOLR2 | 1.2078 | 0.0067 |
| 26 | CHRDL2 | 1.3899 | 0.0025 | 56 | ADGRF5 | 1.2427 | 0.0067 |
| 27 | GPIHBP1 | 1.3642 | 0.0027 | 57 | C8B | 1.4300 | 0.0069 |
| 28 | CHI3L2 | 1.2308 | 0.0027 | 58 | CLDN18 | 1.1506 | 0.0070 |
| 29 | ICAM1 | 1.2041 | 0.0028 | 59 | CSF3R | 1.2466 | 0.0071 |
| 30 | ALOX5 | 1.2404 | 0.0028 | 60 | CCL2 | 1.1720 | 0.0071 |
| No. | Gene | HR | p value | No. | Gene | HR | p value |
| 61 | RASGRP4 | 1.7916 | 0.0072 | 91 | ACSL5 | 1.1733 | 0.0122 |
| 62 | MAMDC2 | 1.2830 | 0.0074 | 92 | ADGRE1 | 1.3593 | 0.0124 |
| 63 | FCGR2A | 1.2471 | 0.0075 | 93 | LRRN4 | 1.3112 | 0.0126 |
| 64 | ADAMTS16 | 1.3474 | 0.0080 | 94 | MUCL3 | 1.7090 | 0.0128 |
| 65 | TNFSF14 | 1.6647 | 0.0083 | 95 | LDB2 | 1.3795 | 0.0132 |
| 66 | CXCR1 | 1.3589 | 0.0084 | 96 | INMT | 1.2004 | 0.0137 |
| 67 | LRRK2 | 1.2127 | 0.0087 | 97 | CYTH4 | 1.2840 | 0.0139 |
| 68 | PLA2G5 | 1.5339 | 0.0087 | 98 | SLC5A8 | 1.3431 | 0.0140 |
| 69 | CCL14 | 1.7907 | 0.0089 | 99 | VSIG4 | 1.1537 | 0.0141 |
| 70 | AGTR2 | 1.2164 | 0.0089 | 100 | PCDHGA12 | 1.5088 | 0.0141 |
| 71 | MSGN1 | 0.6186 | 0.0090 | 101 | CFB | 1.2282 | 0.0142 |
| 72 | DES | 1.1783 | 0.0090 | 102 | CD93 | 1.2289 | 0.0144 |
| 73 | THBS1 | 1.1850 | 0.0091 | 103 | ACVRL1 | 1.3112 | 0.0144 |
| 74 | SSX1 | 1.3822 | 0.0098 | 104 | A2M | 1.1635 | 0.0145 |
| 75 | CASS4 | 1.4712 | 0.0098 | 105 | C4A | 1.2964 | 0.0147 |
| 76 | CNTN6 | 2.1566 | 0.0104 | 106 | MARCO | 1.1218 | 0.0148 |
| 77 | LONRF3 | 1.5015 | 0.0105 | 107 | GPD1 | 1.3525 | 0.0149 |
| 78 | PECAM1 | 1.2514 | 0.0107 | 108 | CCDC177 | 0.5874 | 0.0149 |
| 79 | GGTLC1 | 1.2827 | 0.0107 | 109 | SLC11A1 | 1.2653 | 0.0152 |
| 80 | ABCA6 | 2.0165 | 0.0110 | 110 | RARRES2 | 1.1686 | 0.0154 |
| 81 | ECSCR | 1.3809 | 0.0110 | 111 | IL1RL1 | 1.3939 | 0.0156 |
| 82 | GPRIN3 | 1.4898 | 0.0110 | 112 | CD5L | 1.9618 | 0.0160 |
| 83 | ENPP2 | 1.2322 | 0.0113 | 113 | TNXB | 1.4178 | 0.0160 |
| 84 | KLF2 | 1.2345 | 0.0114 | 114 | REM1 | 1.3792 | 0.0161 |
| 85 | FPR2 | 1.4095 | 0.0117 | 115 | FBP1 | 1.1520 | 0.0163 |
| 86 | SLC39A8 | 1.2241 | 0.0118 | 116 | PTAFR | 1.2383 | 0.0164 |
| 87 | C3 | 1.1651 | 0.0119 | 117 | CD14 | 1.2006 | 0.0165 |
| 88 | MRC1 | 1.1704 | 0.0121 | 118 | FCAR | 1.6205 | 0.0166 |
| 89 | ADH1B | 1.1730 | 0.0121 | 119 | C1QTNF1 | 1.1625 | 0.0166 |
| 90 | LILRB3 | 1.4844 | 0.0121 | 120 | SLC34A2 | 1.0902 | 0.0167 |
| No. | Gene | HR | p value | No. | Gene | HR | p value |
| 121 | STARD8 | 1.4053 | 0.0170 | 151 | C4BPA | 1.0940 | 0.0239 |
| 122 | LILRA5 | 1.2875 | 0.0181 | 152 | GYPC | 1.2067 | 0.0240 |
| 123 | C4B | 1.3103 | 0.0182 | 153 | BIRC3 | 1.1606 | 0.0242 |
| 124 | ADGRE3 | 1.9197 | 0.0189 | 154 | CFAP221 | 1.4590 | 0.0244 |
| 125 | TM6SF1 | 1.4607 | 0.0189 | 155 | CX3CR1 | 1.4367 | 0.0245 |
| 126 | SFTA2 | 1.0940 | 0.0190 | 156 | C5AR1 | 1.2092 | 0.0250 |
| 127 | CCRL2 | 1.3504 | 0.0194 | 157 | SLC22A31 | 1.1329 | 0.0252 |
| 128 | STAB1 | 1.2499 | 0.0197 | 158 | S1PR4 | 1.2478 | 0.0254 |
| 129 | PLA2G2A | 1.1456 | 0.0199 | 159 | TMEM273 | 1.3018 | 0.0261 |
| 130 | CD300LB | 1.6930 | 0.0200 | 160 | NLRP3 | 1.4602 | 0.0262 |
| 131 | ITGAM | 1.2033 | 0.0200 | 161 | SIGLEC5 | 2.1261 | 0.0262 |
| 132 | ZEB2 | 1.3832 | 0.0201 | 162 | ACTA2 | 1.1569 | 0.0264 |
| 133 | CYP1B1 | 1.1584 | 0.0201 | 163 | CD163 | 1.1469 | 0.0266 |
| 134 | FGF7 | 1.2615 | 0.0202 | 164 | PODN | 1.1791 | 0.0273 |
| 135 | ISM2 | 0.6624 | 0.0202 | 165 | CD300C | 1.3046 | 0.0276 |
| 136 | THEMIS2 | 1.2111 | 0.0202 | 166 | COLEC12 | 1.1943 | 0.0279 |
| 137 | LPL | 1.1733 | 0.0205 | 167 | CLEC3B | 1.1842 | 0.0280 |
| 138 | MAP1LC3C | 1.4465 | 0.0213 | 168 | ORM2 | 1.2009 | 0.0289 |
| 139 | GJA5 | 1.1851 | 0.0215 | 169 | PPP1R27 | 0.4079 | 0.0289 |
| 140 | CHI3L1 | 1.1538 | 0.0215 | 170 | PDGFRA | 1.2004 | 0.0290 |
| 141 | RPRM | 0.8110 | 0.0216 | 171 | FCGR3B | 1.2075 | 0.0292 |
| 142 | TNFRSF1B | 1.2093 | 0.0216 | 172 | GIMAP8 | 1.2632 | 0.0316 |
| 143 | ABCA3 | 1.1206 | 0.0220 | 173 | CETP | 1.3806 | 0.0321 |
| 144 | SLCO2B1 | 1.1847 | 0.0221 | 174 | SAA2 | 1.1347 | 0.0329 |
| 145 | VSTM2L | 1.1361 | 0.0226 | 175 | MEF2C | 1.3182 | 0.0330 |
| 146 | MEFV | 1.7719 | 0.0230 | 176 | COMP | 1.1207 | 0.0330 |
| 147 | ICAM2 | 1.2873 | 0.0231 | 177 | ODAPH | 1.4468 | 0.0335 |
| 148 | HAS1 | 1.2974 | 0.0232 | 178 | DPT | 1.1295 | 0.0336 |
| 149 | SUSD2 | 1.1489 | 0.0234 | 179 | FPR1 | 1.1773 | 0.0338 |
| 150 | EMCN | 1.3217 | 0.0236 | 180 | MYBPH | 1.3619 | 0.0339 |
| No. | Gene | HR | p value | No. | Gene | HR | p value |
| 181 | MSR1 | 1.1705 | 0.0348 | 211 | CD300E | 1.2712 | 0.0472 |
| 182 | GCSAML | 2.8218 | 0.0349 | 212 | ADAMTS8 | 1.3044 | 0.0483 |
| 183 | TLR4 | 1.2129 | 0.0350 | 213 | AQP4 | 1.1057 | 0.0489 |
| 184 | P2RX1 | 1.3092 | 0.0355 |  |  |  |  |
| 185 | ITGA8 | 1.2843 | 0.0355 |  |  |  |  |
| 186 | KCNA3 | 1.3163 | 0.0362 |  |  |  |  |
| 187 | SH2B3 | 1.2509 | 0.0362 |  |  |  |  |
| 188 | CYSLTR1 | 1.4517 | 0.0365 |  |  |  |  |
| 189 | CCL3 | 1.2089 | 0.0379 |  |  |  |  |
| 190 | NNMT | 1.1434 | 0.0384 |  |  |  |  |
| 191 | HLA-DRB5 | 1.1133 | 0.0390 |  |  |  |  |
| 192 | SLCO2A1 | 1.1358 | 0.0395 |  |  |  |  |
| 193 | NFATC2 | 1.2611 | 0.0396 |  |  |  |  |
| 194 | SCN7A | 1.3175 | 0.0400 |  |  |  |  |
| 195 | MYO1G | 1.2052 | 0.0411 |  |  |  |  |
| 196 | DEPP1 | 1.1545 | 0.0418 |  |  |  |  |
| 197 | GPSM3 | 1.1892 | 0.0424 |  |  |  |  |
| 198 | AQP1 | 1.1167 | 0.0426 |  |  |  |  |
| 199 | NLRC4 | 1.4963 | 0.0429 |  |  |  |  |
| 200 | KCNMB1 | 1.4132 | 0.0436 |  |  |  |  |
| 201 | PGC | 1.0896 | 0.0437 |  |  |  |  |
| 202 | SYNE1 | 1.4224 | 0.0438 |  |  |  |  |
| 203 | INPP5D | 1.2005 | 0.0439 |  |  |  |  |
| 204 | COL4A3 | 1.3139 | 0.0441 |  |  |  |  |
| 205 | WISP2 | 1.2480 | 0.0449 |  |  |  |  |
| 206 | CD209 | 1.2314 | 0.0449 |  |  |  |  |
| 207 | APBB1IP | 1.2159 | 0.0456 |  |  |  |  |
| 208 | SPI1 | 1.1700 | 0.0460 |  |  |  |  |
| 209 | UGT1A7 | 0.9137 | 0.0468 |  |  |  |  |
| 210 | SIGLEC9 | 1.2982 | 0.0472 |  |  |  |  |

| **Supplementary Table 3. The most important 17 genes in prognostic model in TCGA cohort** | | | |
| --- | --- | --- | --- |
| Factor | HR | 95%CI | P value |
| CCL2 | 1.172 | 1.044-1.316 | 0.0071 |
| CCL21 | 1.1632 | 1.053-1.285 | 0.0029 |
| CSF2 | 1.4028 | 1.144-1.72 | 0.0011 |
| F13A1 | 1.1928 | 1.06-1.342 | 0.0033 |
| FGA | 1.2755 | 1.143-1.423 | <0.0001 |
| HAS1 | 1.2974 | 1.036-1.624 | 0.0232 |
| HPR | 2.2084 | 1.477-3.301 | 0.0001 |
| ISM2 | 0.6624 | 0.468-0.938 | 0.0202 |
| LBP | 1.3631 | 1.161-1.601 | 0.0002 |
| APOH | 1.6357 | 1.326-2.018 | <0.0001 |
| MMP19 | 1.2973 | 1.11-1.516 | 0.0011 |
| MSGN1 | 0.6186 | 0.431-0.887 | 0.009 |
| PTGIS | 1.2778 | 1.091-1.497 | 0.0024 |
| RETN | 1.3818 | 1.171-1.631 | 0.0001 |
| RPRM | 0.811 | 0.678-0.97 | 0.0216 |
| SSX1 | 1.3822 | 1.081-1.767 | 0.0098 |
| C1QTNF1 | 1.1625 | 1.028-1.315 | 0.0166 |
|  |  |  |  |

| **Supplementary Table 4. Univariable and multivariable Cox regression analysis of characteristics with over survival in GSE74777 cohort** | | | | | | |
| --- | --- | --- | --- | --- | --- | --- |
| **Variable** | **Univaruate cox** | | | **Multivariate cox** | | |
|  | **HR** | **CI95%** | ***P* Value** | **HR** | **CI95%** | ***P* Value** |
| Age |  |  |  |  |  |  |
| ≤65 or >65 | 1.267 | 0.688-2.331 | 0.447 | 1.924 | 0.978-3.785 | 0.058 |
| Gender |  |  |  |  |  |  |
| Male or female | 3.13 | 0.755-12.974 | 0.116 | 4.819 | 1.078-21.545 | 0.04 |
| Current smoking |  |  |  |  |  |  |
| Yes or no | 1.646 | 0.887-3.056 | 0.114 | 1.572 | 0.8-3.091 | 0.19 |
| Smoking index |  |  |  |  |  |  |
| ≤30, 30-60, >60 | 1.137 | 0.664-1.945 | 0.641 | 0.905 | 0.456-1.799 | 0.776 |
| T stage |  |  |  |  |  |  |
| 1, 2 or 3 | 1.577 | 0.999-2.49 | 0.051 | 3.044 | 1.202-7.713 | 0.019 |
| N stage |  |  |  |  |  |  |
| 0, 1 or 2 | 1.369 | 0.727-2.577 | 0.331 | 5.291 | 1.529-18.306 | 0.009 |
| TNM stage |  |  |  |  |  |  |
| I or II | 1.367 | 0.744-2.513 | 0.314 | 0.252 | 0.062-1.016 | 0.053 |
| Risk score |  |  |  |  |  |  |
| Increasing | 2.265 | 1.182-4.339 | 0.014 | 2.477 | 1.243-4.937 | 0.01 |
|  |  |  |  |  |  |  |

| **Supplementary Table 5. Univariable and multivariable Cox regression analysis of characteristics with over survival in an independent cohort** | | | | | | |
| --- | --- | --- | --- | --- | --- | --- |
| **Variable** | **Univaruate cox** | | | **Multivariate cox** | | |
|  | **HR** | **CI95%** | ***P* Value** | **HR** | **CI95%** | ***P* Value** |
| Age |  |  |  |  |  |  |
| ≤65 or >65 | 1.089 | 0.372-3.19 | 0.876 | 2.29 | 0.532-9.866 | 0.266 |
| Gender |  |  |  |  |  |  |
| Male or female | 1.14551E+16 | 0-Inf | 1 | / | / | / |
| Current smoking |  |  |  |  |  |  |
| Yes or no | 0.312 | 0.086-1.134 | 0.077 | 1.367 | 0.226-8.277 | 0.734 |
| Smoking index |  |  |  |  |  |  |
| ≤30, 30-60, >60 | 0.696 | 0.321-1.505 | 0.357 | 0.594 | 0.199-1.773 | 0.351 |
| Differentiation |  |  |  |  |  |  |
| High, moderate or poor | 0.696 | 0.321-1.505 | 0.357 | 0.594 | 0.199-1.773 | 0.351 |
| T stage |  |  |  |  |  |  |
| 1, 2 or 3 | 2.069 | 0.868-4.931 | 0.101 | 1.999 | 0.616-6.487 | 0.249 |
| N stage |  |  |  |  |  |  |
| 0, 1 or 2 | 1.727 | 0.966-3.089 | 0.065 | 0.666 | 0.266-1.669 | 0.386 |
| TNM stage |  |  |  |  |  |  |
| I or II | 2.798 | 0.784-9.982 | 0.113 | 2.362 | 0.364-15.303 | 0.367 |
| Risk score |  |  |  |  |  |  |
| Increasing | 5.74 | 1.61-20.466 | 0.007 | 9.854 | 1.67-58.132 | 0.012 |
|  |  |  |  |  |  |  |


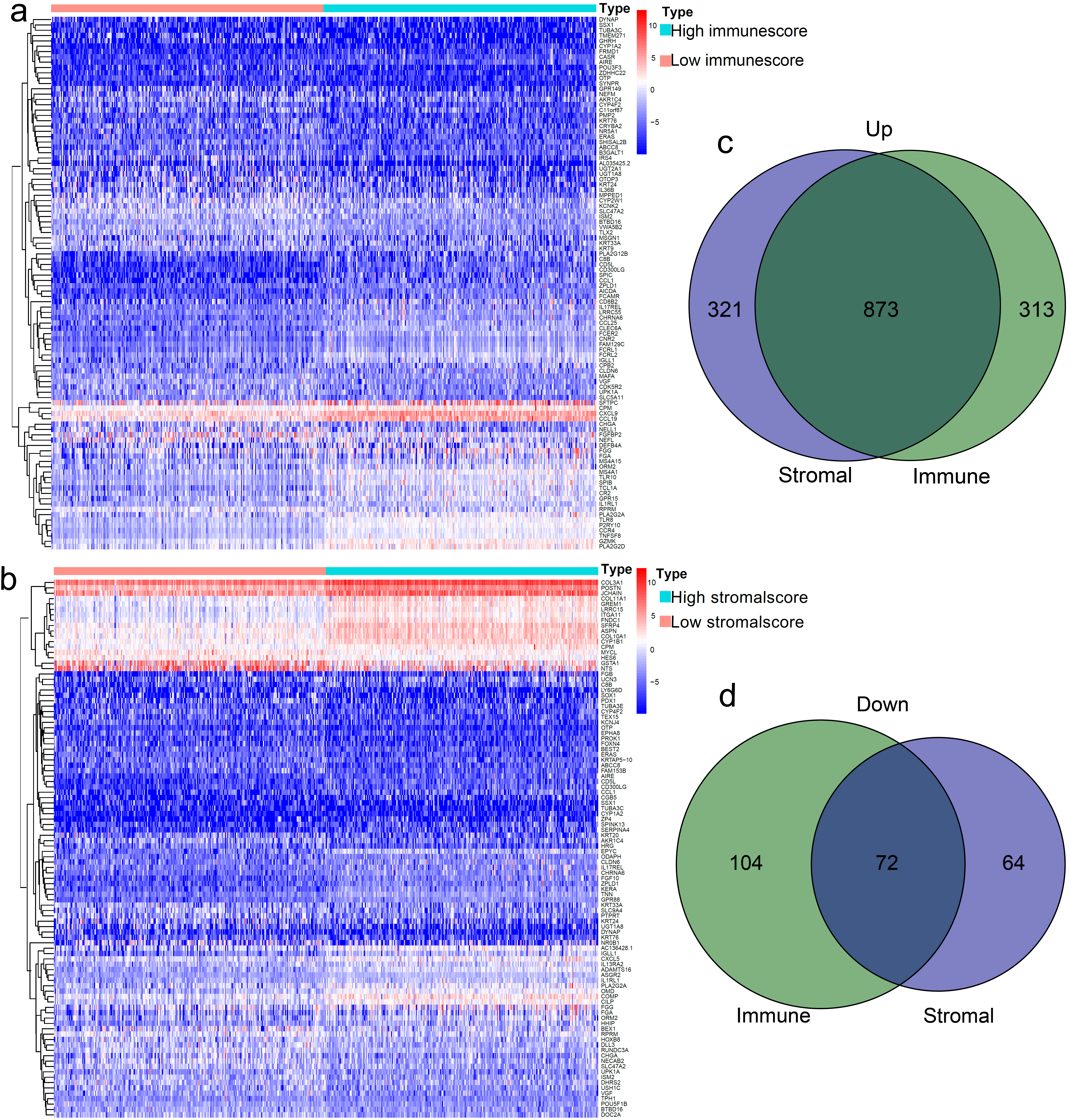


**Supplementary Figure 1**. A total of 945 DEGs generated from high immune (stromal) score and low score groups for LUSC. Heatmap of significantly DEGs based on immune (A) and stromal (B) scores. Venn diagram analysis of high (C) and low (D) expressed genes based on immune and stromal scores.


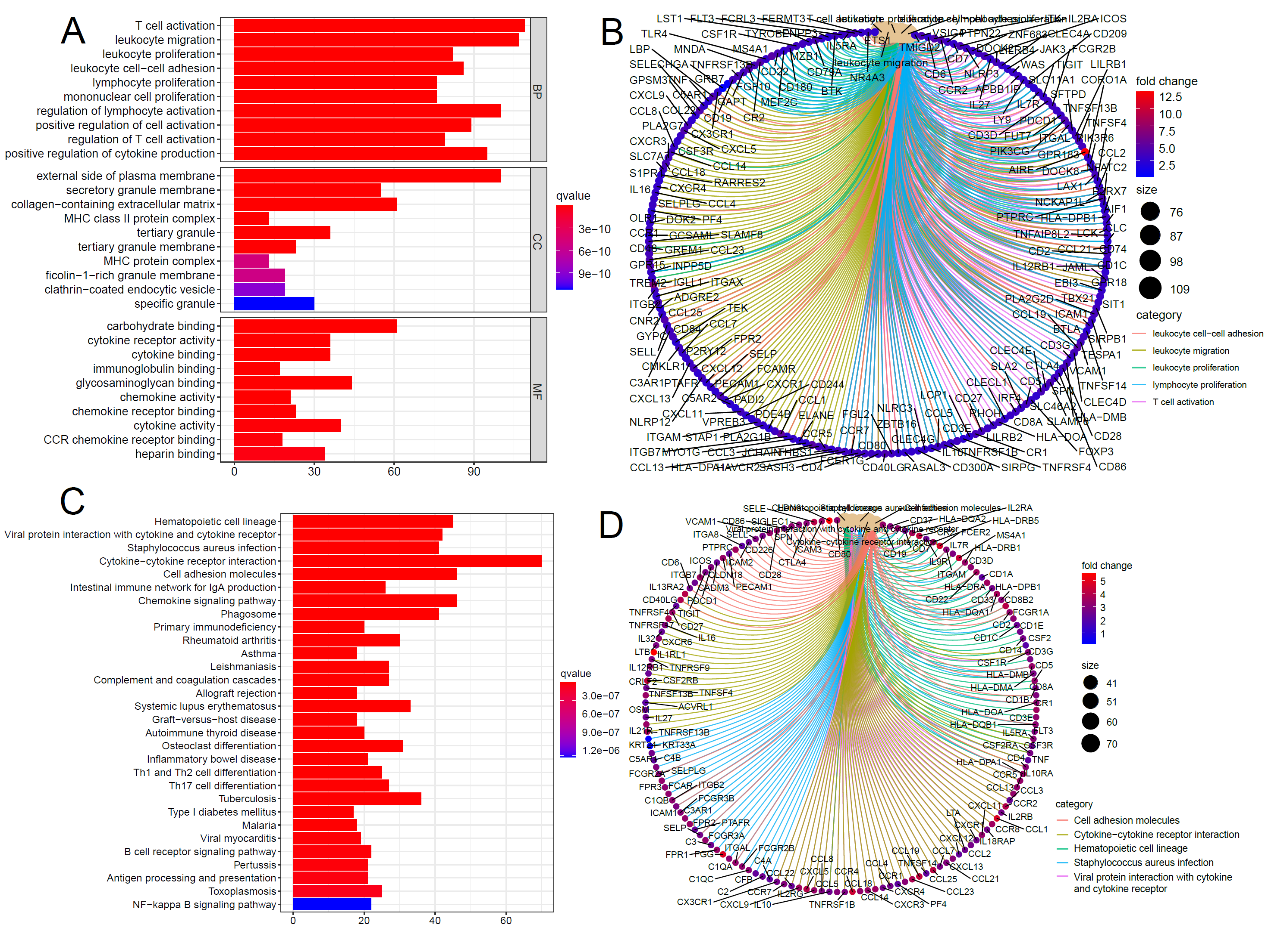


**Supplementary Figure 2.** GO and KEGG enrichment analysis for 945 DEGs. GO analysis (A) of aberrantly expressed genes and CircleMap (B) showing the functional interactions between pathways and genes as extracted from GO. KEGG analysis (C) of aberrantly expressed genes and CircleMap (D) showing the functional interactions between pathways and genes as extracted from KEGG.


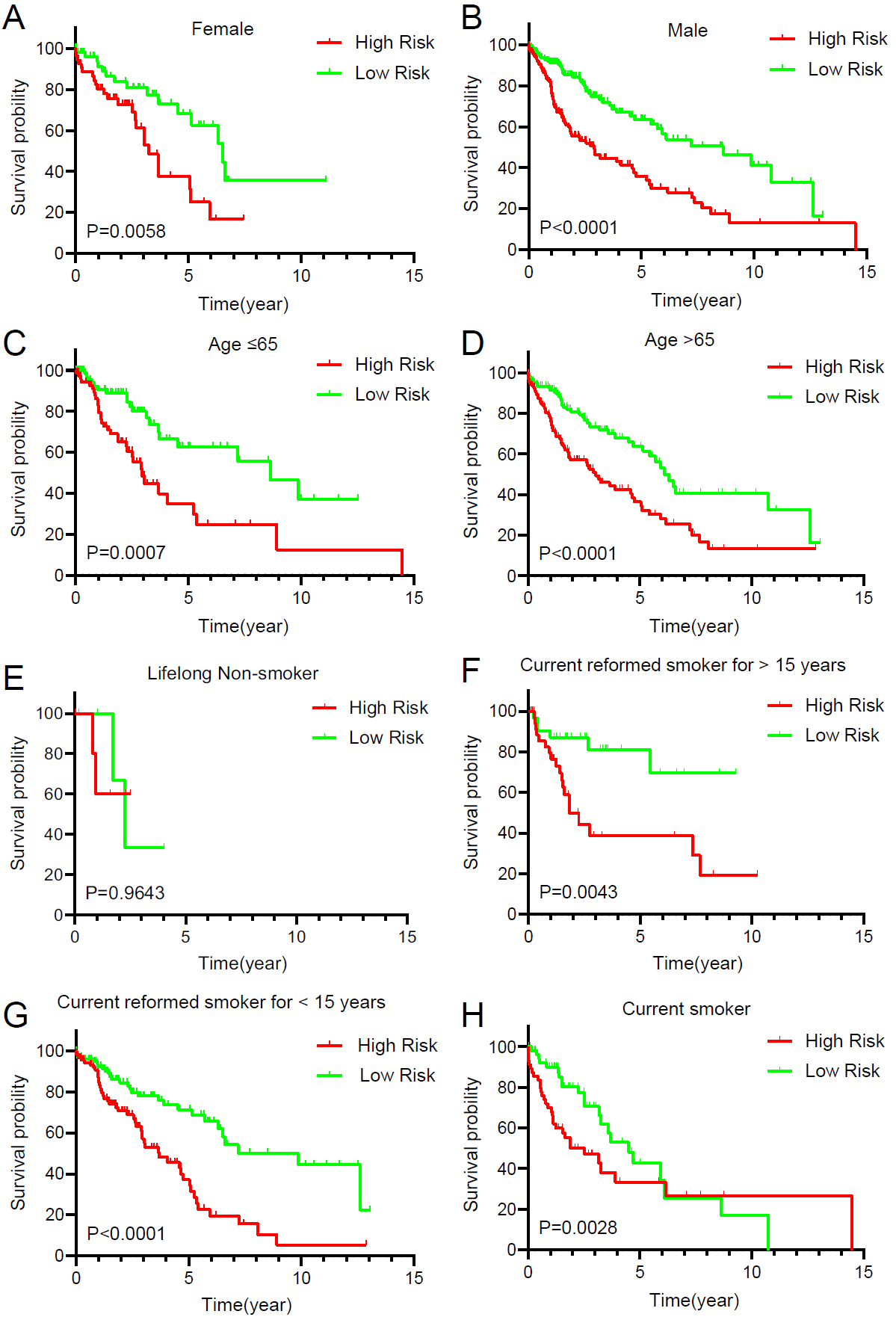


**Supplementary Figure 3.**Validation of predictive value of the immune-related gene signature layered by gender, age, and smoking history in TCGA cohort. Over survival curves for female (A), male (B), age≤65 (C), age>65 (D), lifelong non-smoker (E), current reformed smoker>15 years (F), current reformed smoker<15 years (G), and current smoker (H) based on risk score in early LUSC.


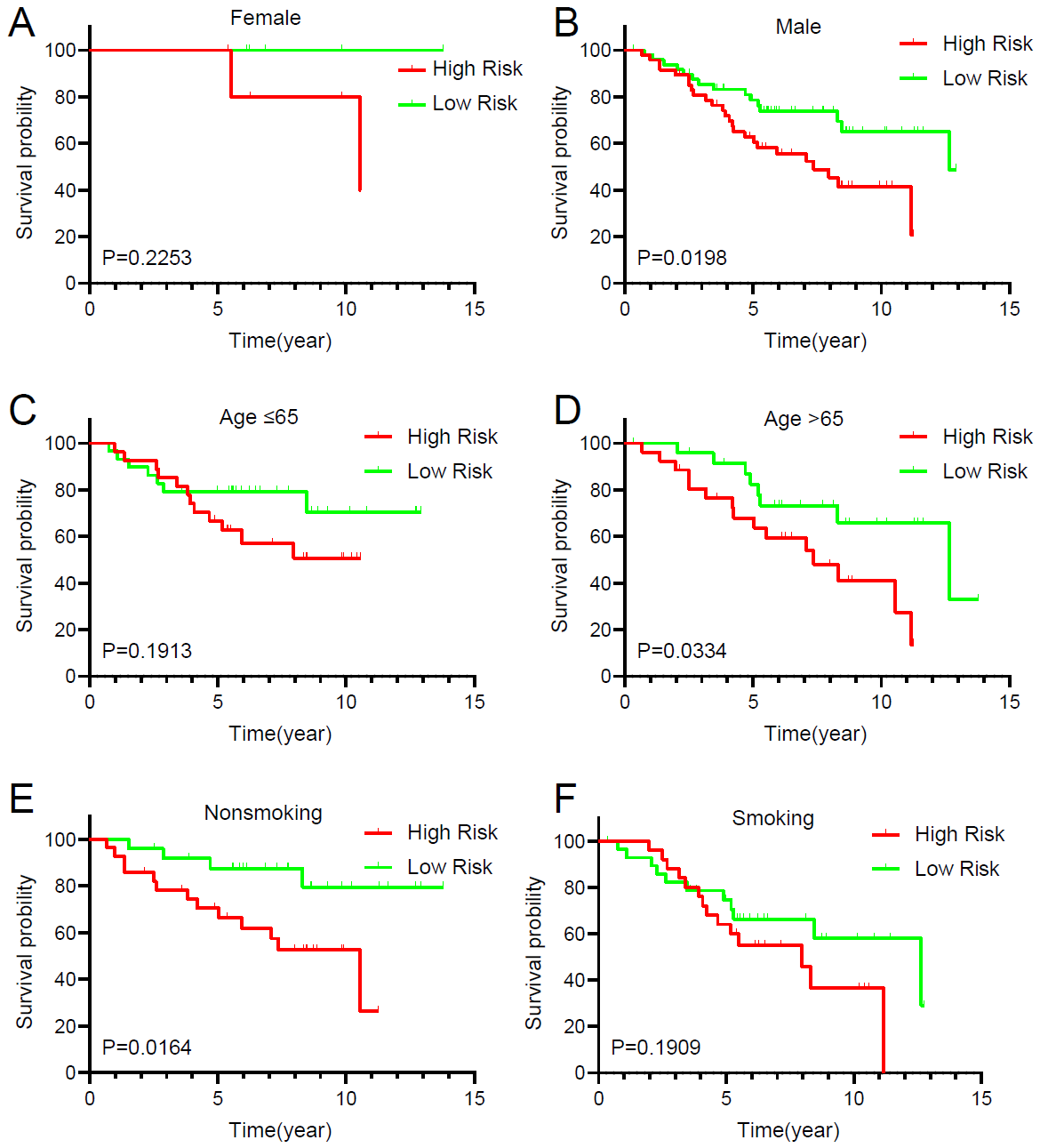


**Supplementary Figure 4.**Validation of predictive value of the immune-related gene signature layered by gender, age, and smoking history in GSE74777 cohort. Over survival curves for female (A), male (B), age≤65 (C), age>65 (D), lifelong non-smokers (E), and smokers (F) based on risk score in early LUSC.


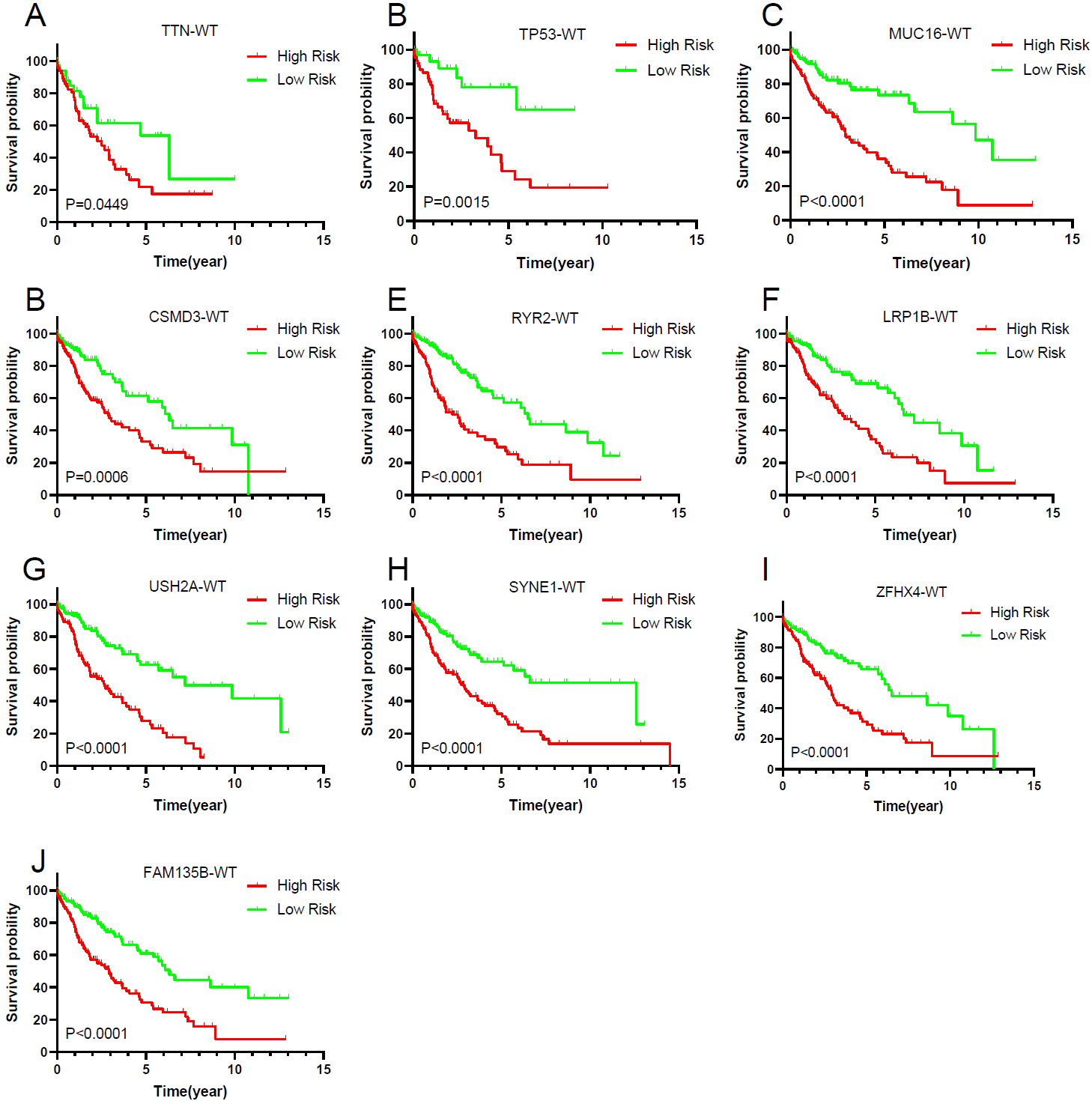


**Supplementary Figure 5.**Validation of predictive value of the immune-related gene signature layered by the 10 most frequently mutated genes. Over survival curves in TTN-WT (A), TP53-WT (B), MUC16-WT (C), CSMD3-WT (D), RYR2-WT (E), LRP1B-WT (F), USH2A-WT (G), SYNE1-WT (H), ZFHX4-WT (I), and FAM135B-WT (J) based on risk score in early LUSC.


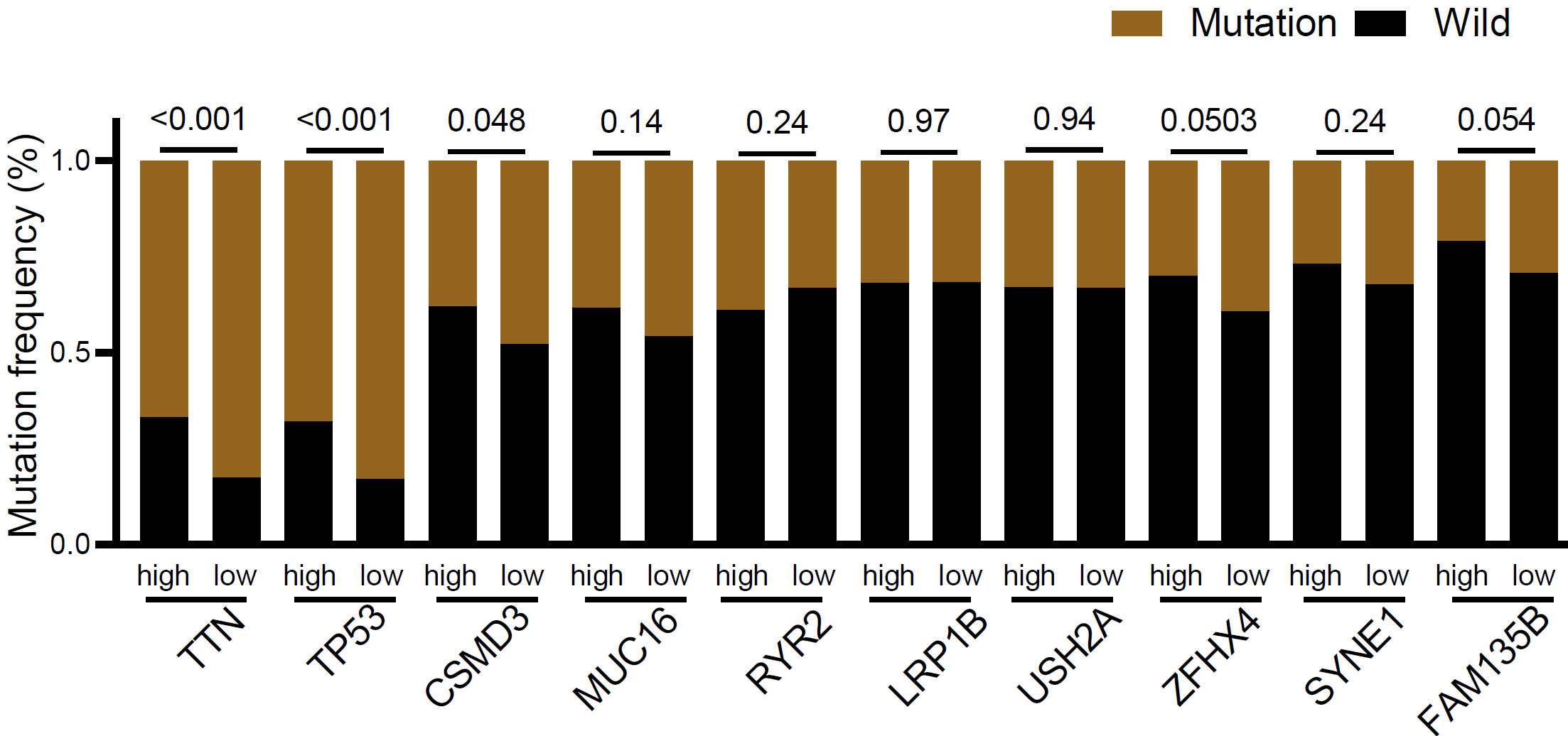


**Supplementary Figure 6**. TTN, TP53, MUC16, CSMD3, RYR2, LRP1B, USH2A, SYNE1, ZFHX4 and FAM135B wild-type and mutation proportions by high risk or low risk.


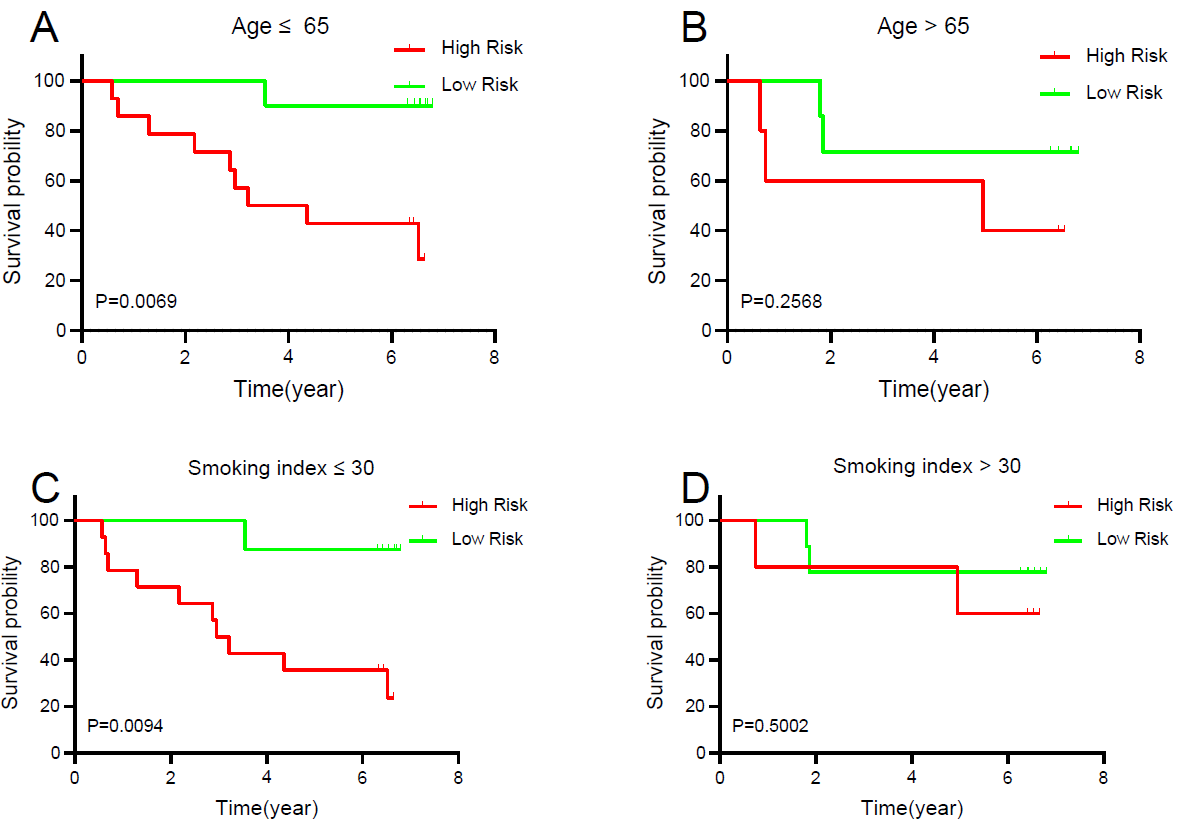


**Supplementary Figure 7.** Validation of predictive value of the immune-related gene signature layered by gender, age, and smoking history in the independent cohort. Over survival curves for age≤65 (A), age>65 (B), smoking index ≤30 (C), and smoking index >30 (D) based on risk score in early LUSC.
